# Academic Background Affects Students’ Confidence and Anxiety Levels about Undergraduate Osteopathic Medical Education

**DOI:** 10.51894/001c.144150

**Published:** 2025-09-30

**Authors:** Vijayashree Jambunathan

**Affiliations:** 1 College of Osteopathic Medicine Michigan State University

## 47

### INTRODUCTION/BACKGROUND

Students entering medical school come in with varied educational experiences that can influence their confidence and anxiety levels. However, those parameters are poorly explored among osteopathic medical students. It is crucial for faculty to consider these differences when designing a medical school curriculum to meet the needs of this diverse group.

### OBJECTIVES/HYPOTHESIS

We hypothesized that incoming osteopathic medical students who did not take certain subjects or had prior negative learning experiences would report higher subject-specific anxiety levels. This study aimed to assess how students perceive their undergraduate and graduate experiences prepared them for medical school. The findings have the potential to shape quality improvement initiatives for the curriculum at MSUCOM.

### METHODS

This cross-sectional study surveyed incoming MSUCOM students (n=194) using an anonymous questionnaire during orientation week (IRB approval: STUDY00010320). The survey included questions about undergraduate major, post-undergraduate studies, science course load, confidence and anxiety, and negative learning experiences. Data were analyzed using Chi-square, Fisher’s exact test, and odds ratios.

### RESULTS

Of the respondents, 81% reported a medium undergraduate science load (2-3 science classes/semester), with about two-thirds feeling somewhat confident about starting medical school. Students who took anatomy, genetics, microbiology, and physiology as an undergraduate had significantly lower odds of anxiety about those subjects compared to students who had not (p<0.05). On the other hand, having taken either immunology or pharmacology/toxicology did not reduce students’ anxiety levels. Approximately 31% of students reported a negative learning experience in biochemistry, and those students had 9 times higher odds of anxiety (OR=9.3, 95% CI: 4.2-19.5, p<0.001). As hypothesized, students with negative learning experiences reported significantly higher anxiety levels (p<0.05) for those subjects entering medical school.

### DISCUSSION/CONCLUSIONS

Undergraduate and graduate experiences significantly influence incoming medical students’ confidence and anxiety levels. Some subjects, like biochemistry, immunology, and pharmacology/toxicology, are more likely to contribute to student anxiety. Future surveys will assess how students’ perceptions evolve during medical school. Future work will address how to support osteopathic medical students in these high-anxiety subjects and ease the medical school transition.

